# Vibration transmittance measures sternotomy stability – a preliminary study in human cadavers

**DOI:** 10.1186/s13019-018-0823-5

**Published:** 2019-01-07

**Authors:** Juha Hautalahti, Atte Joutsen, Sirkka Goebeler, Tiina Luukkaala, Jahangir Khan, Jari Hyttinen, Jari Laurikka

**Affiliations:** 1 Department of Cardiothoracic Surgery, Tampere Heart Hospital Co., Ensitie 4, FI-33520 Tampere, Finland; 2 Faculty of Medicine and Life Sciences, University of Tampere, Arvo Ylpön katu 34, FI-33520 Tampere, Finland; 3 BioMediTech Institute and Faculty of Biomedical Sciences and Engineering, Tampere University of Technology, Arvo Ylpön katu 34, FI-33520 Tampere, Finland; 4 Forensic Medicine, National Institute for Health and Welfare, Biokatu 16, O-building, FI-33520 Tampere, Finland; 5 Science Center, Tampere University Hospital, Teiskontie 35, FI-33521 Tampere, Finland; 6 Health Sciences, Faculty of Social Sciences, University of Tampere, Arvo Ylpön katu 34, FI-33520 Tampere, Finland

**Keywords:** Sternum, Sternotomy, Wound healing, Postoperative complications, Integrity, Vibration transmittance, Electronics, 10.050 Cardiac / Basic Science, 20.030 Thoracic / Pleura and Chest wall

## Abstract

**Background:**

Stability is essential for the normal healing of a sternotomy. Mechanical vibration transmittance may provide a new means of early detection of diastasis in the sternotomy and thus enable the prevention of further complications. We sought to confirm that vibration transmittance detects sternal diastasis in human tissue.

**Methods:**

Ten adult human cadavers (8 males and 2 females) were used for sternal assessments with a device constructed in-house to measure the transmittance of a vibration stimulus across the median sternotomy at the second, third, and fourth costal cartilage. Intact bone was compared to two fixed bone junctions, namely a stable wire fixation and an unstable wire fixation with a 10 mm wide diastasis mimicking a widely rupturing sternotomy. A generalized Linear Mixed Model with the *lme* function was used to determine the ability of the vibration transmittance device to differentiate mechanical settings in the sternotomy.

**Results:**

The transmitted vibration power was statistically significantly different between the intact chest and stable sternotomy closure, stable and unstable closure, as well as intact and unstable closure (*t*-values and *p*-values respectively: *t* = 6.87, *p* < 0.001; *t* = 7.41, *p* < 0.001; *t* = 14.3, *p* < 0.001). The decrease of vibration transmittance from intact to stable at all tested costal levels was 78%, from stable to unstable 58%, and from intact to unstable 91%. The vibration transmittance power was not statistically significantly different between the three tested costal levels (level 3 vs. level 2; level 4 vs. level 2; level 4 vs. level 3; *t*-values and *p*-values respectively *t* = − 0.36, *p* = 0.723; *t* = 0.35, *p* = 0.728; *t* = 0.71, *p* = 0.484).

**Conclusions:**

Vibration transmittance analysis differentiates the intact sternum, wire fixation with exact apposition, and wire fixation with a gap. The gap detection capability is not dependent on the tested costal level. The method may prove useful in the early detection of sternal instability and warrants further exploration.

## Background

Median sternotomy is the most common access in open heart surgery. The estimated annual number of sternotomies in Finland is approximately 3800 per a population of 5.5 million, and the equivalent figure in the USA is about 500,000 per a population of 323 million [1, 2]. The disruption of sternal steel wire fixation occurs when the separating forces exceed the mechanical holding properties of the closure. The reported incidence of post-sternotomy instability ranges from 0.39 to 1.6% up to 6 months postoperatively [3–5]. Even minor instability may cause subjective symptoms and can progress to complete wound disruption often complicated by deep sternal wound infection. Early detection of sternal instability may enable preventative measures such as the use of supportive vests as well as surgical exploration and re-fixation of the wound that may prevent the later more severe complications [6–8]. Risk factors for instability include numerous patient factors, as well as operative and postoperative variables [9–18].

The mechanical stability of the bone fracture or osteotomy is crucial for the formation of a callus and the maturation phase of the lamellar bone [19]. Stability is also essential for successful bone healing after sternotomy [9, 10], but it is fairly difficult to measure. Detection of a failing sternotomy is commonly done by manual palpation, which is a subjective method and prone to misinterpretations [20]. Computed tomography offers only indirect information on sternal stability, and signs of sternal bone healing appear months after surgery [21–23], whereas sternal instability leading to wound disruption and infection most often occurs within the first month of the operation [3]. Ultrasound has been used to evaluate sternal nonunion and gross instability years after surgery [24], but the method obviously carries a risk of contaminating the wound in the immediate postoperative period.

Vibration transmittance has been used to assess bone fractures and bone density as well as dental and orthopedic implant stability [25–27]. With fixed input excitation, i.e. power emitted by the vibration actuator, the detected power measured by the accelerometer sensor acts as a measure of the mechanical integrity of the studied object. We have reported vibration transmittance as a tool to assess and follow postoperative sternal stability [28], and we postulated that non-invasive detection of early sternotomy diastasis could be possible. The aim of the present study was to describe vibration transmittance in the human sterna in a cadaver model. The hypothesis of the study was that vibration transmittance assessment could be used to differentiate intact, surgically fixated, and unstable sterna.

## Methods

**Fig. 1:**
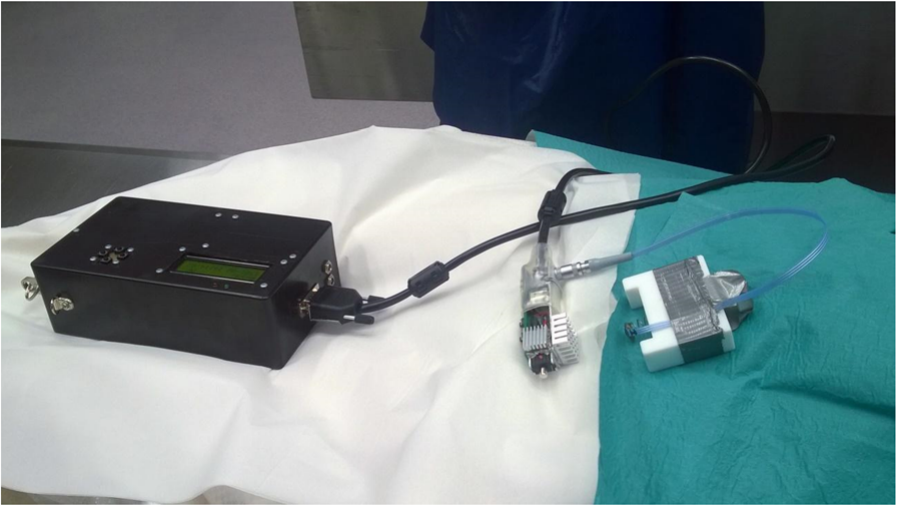
Vibration transmittance measurement device. Main unit, actuator and sensor (from left to right)

**Fig. 2:**
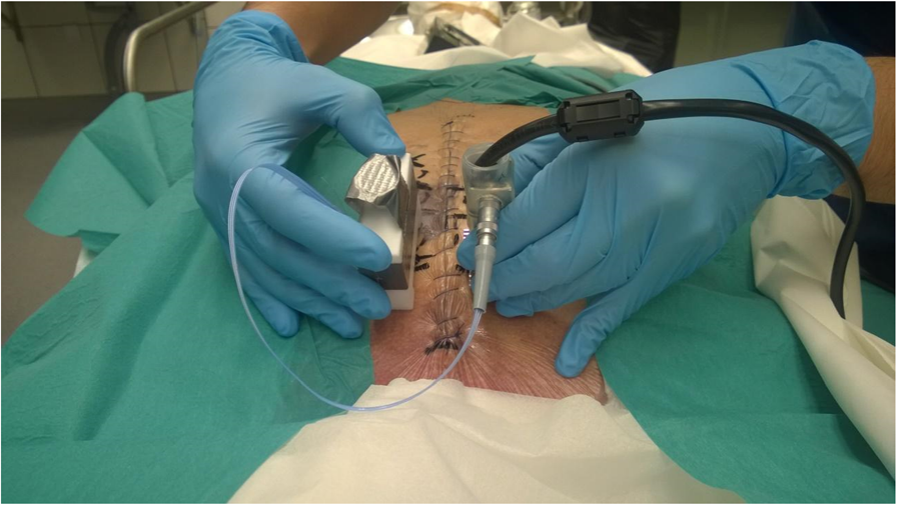
Measurement of vibration transmittance. The sensor and actuator are held by hand and placed perpendicular in relation to the chest surface

**Fig. 3:**
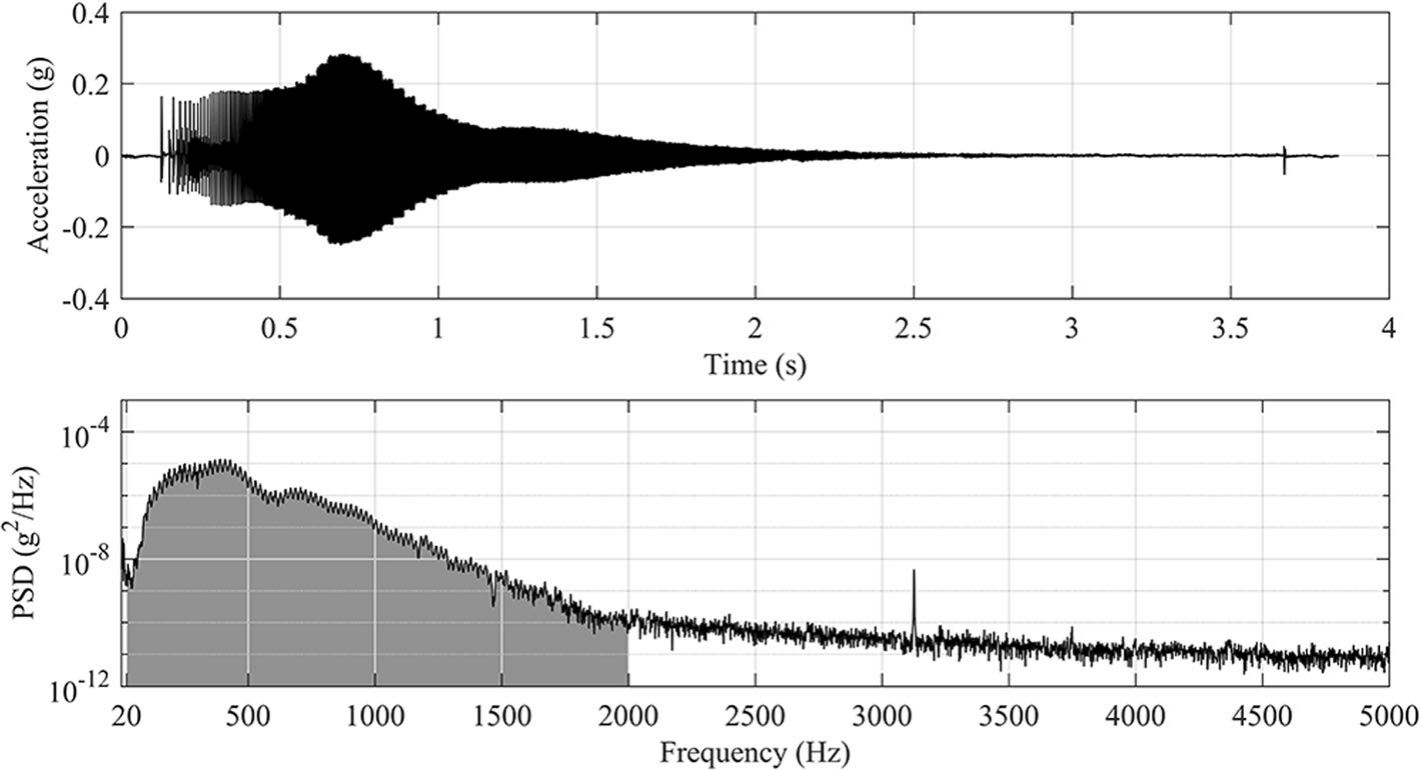
An example of a vibration sweep output. Above: a vibration sweep output picked up by the accelerometer sensor according to the time. Below: the same vibration sweep results according to the measured frequencies. The gray-shaded area under the curve represents the total power integral in the 20-2000 Hz band

The study population consisted of 10 human cadavers. The age, height, weight, post-mortem time, soft tissue thickness, sternum thickness and presternal soft tissue temperature were recorded in each case, as shown in Table 1. The vibration transmittance measurement device used in this study was the third-generation version of an in-house-constructed, embedded vibration measurement system consisting of an actuator, a sensor, and a main controller unit. The device is compact, portable, and battery driven (Fig. 1). The actuator introduces a vibration stimulus to the tissue, sweeping a band of 20–2000 Hz over 3.7 s. The sensor consists of an accelerometer that records the transmitted vibration at a 10 kHz sample rate. During measurements, the actuator and sensor are held manually on the chest surface (Fig. 2). Figure 3 shows a typical vibration transmittance graph.

**Table 1:** Descriptive characteristics of study cadavers (8 males, 2 females)

	Median	Range
Age (years)	63	43–78
Height (cm)	171	157–187
Weight (kg)	79	51–100
BMI (kg/m^2^)	27.0	20.7–28.6
Temperature^a^ (°C)	19	16–21
Post-mortem interval (days)	5	2–7
Soft tissue thickness^b^ (mean, mm)	12.5	4–19
Sternum thickness^b^ (mean, mm)	13	10–21

^a^Presternal soft tissue temperature at the end of the study session

^b^Measured at the 2nd, 3rd, and 4th costal level

The skin was covered with an adhesive plastic film. Three measurement levels were used: on the top of the second, third, and fourth costal cartilage. The vibration actuator was positioned 3 cm to the right and the sensor 3 cm to the left of the midline. The vibration transmittance measurements were first performed on an intact chest, followed by unstable chest, and finally in a closed chest, five times at each setting and costal level. In the unstable chest model, a standard median sternotomy was conducted, followed by the insertion of six single no. 7 sternal wires (Ethicon, NJ, USA), and by leaving a 10 mm space between the sternal halves. Ultrasound gel was used to fill the gap and to mimic fluid accumulation. As there was a tendency for the gap to close in rigid cadaver chests, lateral rib traction was used to maintain the gap without obstructing the measurements. The chest was then tightly closed and the soft tissues sutured in two layers for the final measurements.

The data were processed off-line on a personal computer using routines implemented in a MATLAB (Mathworks, MA, USA) scientific computing environment. Total vibration power (g^2^) in the 20-2000 Hz band was calculated for each measurement and logarithmic (ln) transformation was applied before statistical testing, since the raw data were not normally distributed. A generalized Linear Mixed Model with the *lme* function was used to determine the ability of the vibration transmittance device to differentiate mechanical settings in the sternotomy regardless of the costal level. The mean response was modelled as a linear combination of the population characteristics shared by all individuals (fixed effects), and subject-specific effects unique to a particular cadaver constituted the random effects. Three different mechanical settings and three costal levels were modelled as fixed effects and the number of repeated measurements per each cadaver constituted a potential source of variation and was included as random effects in the model [29]. The generalized linear mixed model analyses were performed with the Statistical Package R version 3.3.0 package lme4 (The R Foundation, www.r-project.org). All *p-*values are two-tailed. A *p*-value less than 0.05 was considered statistically significant.

The study was conducted in accordance with the Declaration of Helsinki. The study protocol was approved by the Regional Ethics Committee (Approval number ETL R14131). The relatives of each study subject were contacted by the coroner (SG) and their consent sought before inclusion in the study.

## Results

**Fig. 4:**
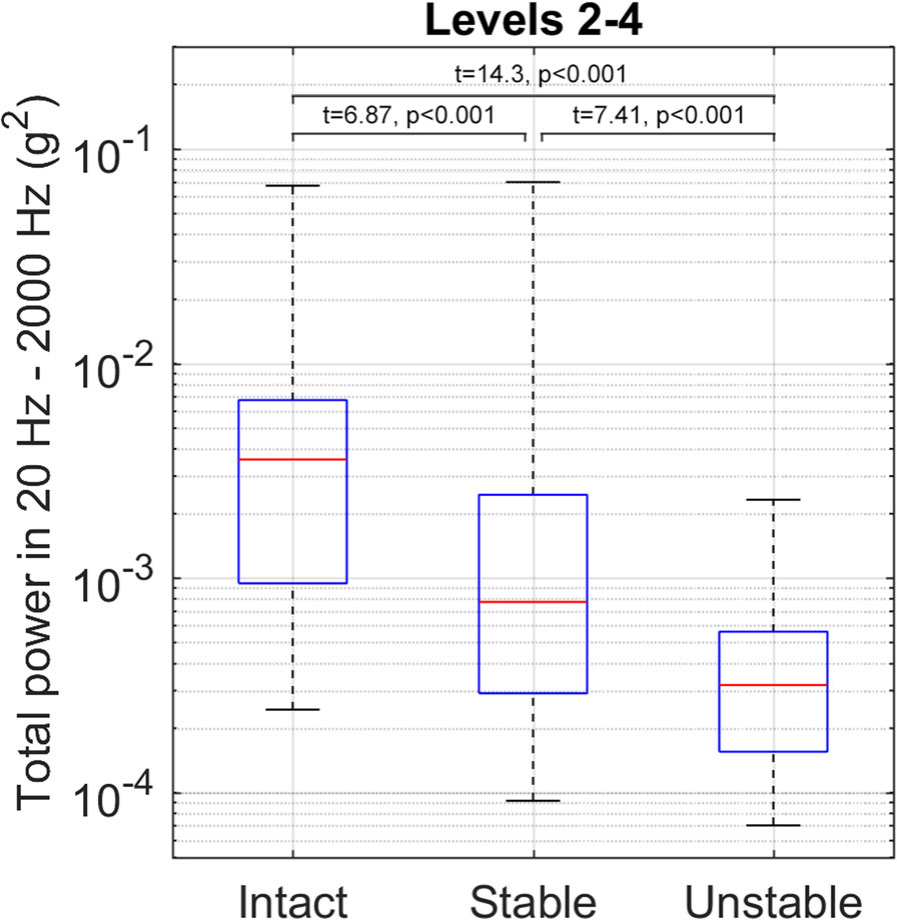
The combined results at the 2nd, 3rd and 4th rib. Stable fixation indicates the sternum is attached tightly with 6 steel wires and unstable fixation indicates a 10 mm distance between the sternal halves

The measured vibration transmittances are given in Tables 2 and 3 shows the total amount and percentage of vibration transmittance reduction at different costal levels and each state of the sternum, calculated from the medians of raw data. There were clear and statistically significant differences between intact, stable, and unstable sterna, and the reduction in vibration transmittance was able to differentiate between stable and unstable sternum, as shown in Fig. 4. There were no statistically significant differences in the vibration transmittance between the three costal levels that were tested, which is presented in Table 4. The soft tissue thickness in cadavers showed a moderate inverse correlation to the transmitted vibration power when the raw data of all tested levels were analysed in the intact sterna (Spearman’s nonparametric rho = − 0.478).

**Table 2:** The median measured vibration transmittance levels (× 10^− 5^) in the 20 Hz – 2000 Hz band (g^2^)

	Intact		Stable		Unstable	
	Median	(range)	Median	(range)	Median	(range)
2^nd^ rib	416	(24–2094)	99	(13–2312)	38	(7–118)
3^rd^ rib	346	(36–6756)	50	(12–7025)	32	(9–78)
4^th^ rib	305	(65–1603)	81	(9–834)	29	(9–233)
Levels combined	357	(24–6756)	77	(9–7025)	32	(7–233)

Stable fixation indicates the sternum is attached tightly with 6 steel wires and unstable fixation indicates a 10 mm distance between the sternal halves

**Table 3:** The change in the median vibration transmittance between different states of the sternum (× 10^− 5^ g^2^)

	At 2^nd^ rib	At 3^rd^ rib	At 4^rd^ rib	Levels combined
Intact → Stable	− 317 (− 76%)	− 296 (− 86%)	− 224 (− 73%)	− 280 (− 78%)
Stable → Unstable	−61 (− 61%)	− 18 (− 37%)	−52 (− 64%)	− 45 (− 58%)
Intact → Unstable	− 378 (− 91%)	− 314 (− 91%)	− 276 (− 91%)	− 325 (− 91%)

**Table 4:** Vibration transmittance power comparisons between the three tested costal levels. Statistical comparisons were calculated from ln-transformed data using a generalized Linear Mixed Model analysis

	t-value	*p*-value
3^rd^ vs 2^nd^	−0.36	0.723
4^th^ vs 2^nd^	0.35	0.728
4^th^ vs 3^rd^	0.71	0.484

## Discussion

Current clinical practice is lacking proper methods for sternal stability assessment. This motivated us to test vibration transmittance as an objective, repeatable, fast, inexpensive and noninvasive tool for the sternotomy dehiscence detection. This method lacks the risks of ionizing radiation and intravenous contrast medium, which are obvious problems when fairly expensive computed tomography is used. Our main finding was that vibration transmittance can be applied as a measure of postoperative sternal integrity.

With the transmittance measurement we achieved diagnostic separation between the intact and split sterna, especially when there was a gap. While the magnitude of vibration transmittance reduction was greatest between an intact sternum and a loose closure with a gap, the method appeared to be able to differentiate between a tightly closed and an unstable sternum as well, the situation with the most clinical relevance. The analyzed 20–2000 Hz band was the same as the stimulation band. Generally, in the low frequency vibration transmittance analysis, the optimal frequency band may differ depending on the device function and measured objects. A deeper search could have also revealed other sub-bands that in turn might better discern sternal stability, however, it was not the goal to optimize the test performance on the cadavers, as the intended use is in postoperative patients recovering from surgery. Indeed, in the preceding study we discovered that the optimal band to depict the stability of normally healing sternotomy is 600–1500 Hz in clinical patients [28], probably because the patients studied were normothermic and recovering from a cardiopulmonary bypass with tissue swelling. The reduction in the vibration transmission caused by the sternotomy was roughly the same even when the explored frequency band was different from our preceding study. As the device technology development is still in the early phase, a10 mm gap resembling widely broken sternum fixation was chosen as a pathological reference. The gap detection capability in the current study was not dependent on the tested costal level which is a sound and clinically relevant finding.

The main problem of in vivo vibration measurements of bone arises from the damping effects of skin and soft tissues, especially in obese patients [25]. A minor damping effect of soft tissues was seen in our series. It should be noted, however, that this series was a proof of concept and feasibility test, and we intentionally chose cadavers with near normal body mass index to exclude the possible bias caused by excessive soft tissue damping. The role soft tissue thickness needs to be confirmed in a larger series. The cadaver model is a valuable platform for vibrational sternotomy stability studies because it offers opportunities to objectively settle different mechanical conditions in the human thorax for serial, repeated measurements. We acknowledge that e.g. the tissue rigidity and the temperature in the cadavers differ from living tissue, and this may somewhat limit the translation of our findings to clinical postoperative patients. The accuracy of the vibration transmittance method to detect smaller bone diastases, as well as characterizing the different stages of normal and disturbed healing in the sterna, requires further clinical study.

## Conclusions

In conclusion, vibration transmittance analysis was applicable and able to differentiate intact sterna, tightly wire-fixed sterna, and sterna with diastasis in the wire-fixed bone halves. The gap detection capability is not dependent on the tested costal level. The concept was proven to be promising, as it offers a tool for the earlier detection of diastasis in sternotomies, which potentially enables the prevention of sternotomy wound complications.
